# Assessing Individual Competency Differences Between Third- and Fourth-Year Medical Students Using the National Clinical Assessment Tool for Medical Students in the Emergency Department

**DOI:** 10.7759/cureus.56486

**Published:** 2024-03-19

**Authors:** Siddhant Kumar, Elizabeth H Jensen, Susan Watts, Michael Parsa

**Affiliations:** 1 Emergency Medicine, Texas Tech University Health Sciences Center El Paso, El Paso, USA

**Keywords:** national clinical assessment tool, medical student assessment, medical student clerkship, medical student performance, medical education, emergency medicine, ncat-em

## Abstract

Medical students rotating through emergency departments as part of their clinical education are typically evaluated using an on-shift evaluation tool. The National Clinical Assessment Tool for Medical Students in the Emergency Department (NCAT-EM) is the current standard of evaluation for medical students in the emergency department, regardless of level of training. This study aims to evaluate whether the NCAT-EM can detect differences in skill levels between third-year medical students (MS3s) and fourth-year medical students (MS4s) rotating at a level 1 trauma center and teaching institution. These authors hypothesized that MS4s should outperform MS3s across all assessment domains given their additional training. A total of 930 performance evaluations were gathered for MS3 and MS4 rotating between May 2022 and June 2023. There were 321 evaluations of MS3s and 609 evaluations of MS4s. Across the six assessment domains, MS4s had statistically significant higher performances in two domains - namely emergency recognition and management (fully entrustable: 37.4% vs. 23.8% (p = 0.03)) and communication (fully entrustable: 46.2% vs. 33.6% (p = 0.03)). These findings indicate that the use of the NCAT-EM at this institution reliably differentiated between MS3s and MS4s in these two assessment domains. There were trends suggesting MS4s outperform MS3s in the other four domains, which did not rise to the level of statistical significance, but are consistent with prior validation studies of the NCAT.

## Introduction

As an inherently dynamic and increasingly growing specialty, emergency medicine has had a highly variable role in the education of medical students [1]. According to one study, just over half of allopathic medical schools in the U.S. require some clerkship in the emergency department [2]. These required clerkships may occur in the third or fourth year and students can expect to be evaluated by both resident and attending physicians [3,4]. Historically, individual institutions used their own assessment tools for on-shift evaluations, which have not been validated externally and may lead to issues comparing scores from different institutions when it comes time for students to apply for residency [5,6]. With such a wide variation in the approach to assessing medical students within the specialty of emergency medicine, attempts have been made to standardize various aspects of the evaluation process, particularly in required clerkships and audition rotations.

The most notable tool developed for clerkship evaluations has been the National Clinical Assessment Tool for Medical Students in the Emergency Department (NCAT-EM) in 2016. This assessment tool was developed during a consensus conference attended by emergency medicine educators from multiple institutions during the Council of Residency Directors in Emergency Medicine (CORD-EM) Academic Assembly, with the goal to provide clerkship directors with a standard assessment tool with broad utilization for students working in the emergency department. A key portion of the NCAT-EM is the six assessment domains with anchors that encompass the first page of the two-page assessment tool [7,8].

These six assessment domains are as follows: focused history and physical exam skills (H&P), ability to generate a prioritized differential diagnosis (DDx); ability to formulate a plan - including diagnostic, therapeutic, and disposition plans (formulate plan), observation, monitoring, and follow-up (observation/monitoring), emergency recognition and management (emergency recognition and management), patient- and team-centered communication (communication).

Students are graded as “pre-entrustable, mostly entrustable, fully entrustable, and outstanding.” Please see the figure for the anchoring statements that provide further details about the performance characteristics linked to each entrustable level for each domain (Appendix - NCAT-EM).

Professionalism is also evaluated but is assessed separately from these six domains. At the end of the assessment, students are ranked as lower 1/3, middle 1/3, top 1/3, or exceptional, meaning top 10% [8].

Following its first year of use, the original authors collected data from 13 sites across 12 states to evaluate the use of the tool [9]. They found that there was an overall positively skewed score distribution, which is not unusual for EM assessment tools [10]. In that same year, Emery et al. evaluated how often professionalism flags were being identified on the NCAT-EM and found that such flags were uncommon but did correlate to overall lower scores when present [11]. Neither study found significant score differences between genders, however, Hiller et al. [9] did find that fourth-year medical students (MS4s) typically scored higher than third-year medical students (MS3s). This was not unexpected; if the assessment tool accurately gauges skills, more advanced learners should score higher, however, there was significant variability across sites regardless of whether students were MS3s or MS4s. Such results make it difficult to determine whether these were true differences in skill level identified by the assessment tool or simply differences in institutional expectations. To our knowledge, no study has been done to compare scores between third- and fourth-year students within the same institution, undergoing the same curriculum at different educational stages. This past academic year, Paul L. Foster School of Medicine at the Texas Tech University Health Sciences Center El Paso, transitioned the EM clerkship from a required fourth-year rotation to a required third-year rotation, creating a unique opportunity in which both third- and fourth-year students were rotating and undergoing evaluation in the same emergency department at the same time. This study aims to evaluate whether the NCAT-EM is effective at determining differences in skill levels between MS3 and MS4 rotating at a level 1 trauma center and teaching institution. These authors hypothesized that MS4s should outperform MS3s across all assessment domains.

## Materials and methods

The Texas Tech University Health Sciences Center El Paso Institutional Review Board issued approval E23029. Throughout the academic year (May 2022-June 2023), NCAT-EM end-of-shift evaluations for both MS3 and MS4 were collected as part of the medical school curriculum rather than for this study.

The data from these evaluations was extracted into a spreadsheet for analysis. To classify a student’s level of clinical experience, an unblinded team member created a variable called "months since the start of year 3" for each student evaluation. The starting date of the academic year (May 1, 21 for MS4; May 1, 22 for MS3) was subtracted from the evaluation date. Thus, beginning MS3 students would have values indicating fewer months of clinical experience than MS4 students. The data were then de-identified and analyzed to determine whether there was a correlation between the mean scores of each performance metric and level of clinical experience. A total of 930 performance evaluations were collected: 321 evaluations for 49 MS3s who rotated in the emergency department and 609 for 149 MS4s. The MS4s had more opportunities to request evaluations since they rotated for four weeks while the MS3 rotation was two weeks.

Data analyses were performed using Stata12 (StataCorp. 2011. Stata Statistical Software: Release 12. College Station, TX: StataCorp LP).

For each of the six domains, the proportion of MS3s and MS4s at each level of entrustability was calculated and compared using chi-square analysis. Regression analysis was conducted to determine the correlation between the level of entrustability and months since the beginning of the third year.

A similar technique to one used by Hiller et al. [9] was performed on our data set. “Pre-entrustable” was assigned a value of 25%, “mostly entrustable” 50%, “fully entrustable” 75%, and “outstanding” 100%. In our study, “pre-entrustable” was assigned a value of 1, “mostly entrustable” 2, “fully entrustable” 3, and “outstanding” 4. The average score of all MS3s was compared to that of all MS4s across all evaluations.

## Results

**Figure 1:**
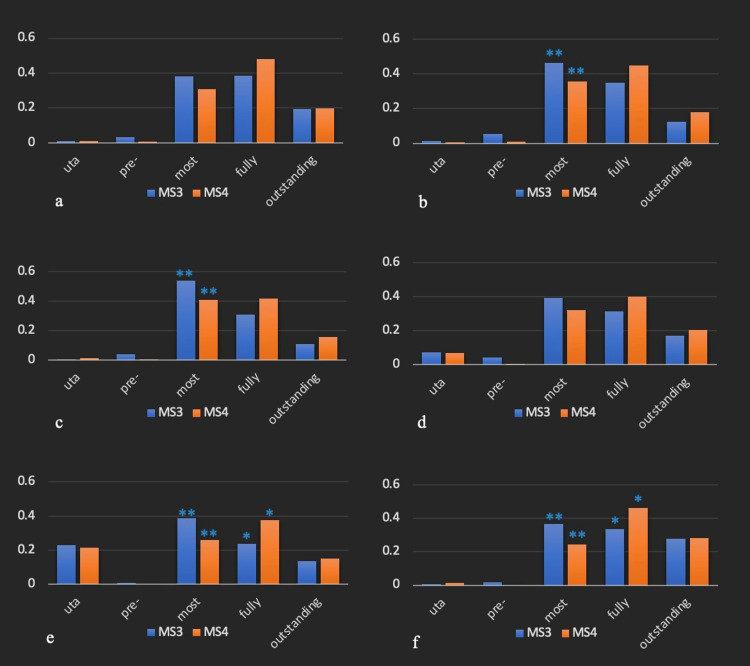
Chi-square analysis of third-year (MS3) vs. fourth-year (MS4) medical student performance a. Focused history and physical exam skills; b. Ability to generate a prioritized differential diagnosis; c. Ability to formulate a plan (diagnostic, therapeutic, disposition); d. Observation, monitoring, and follow-up; e. Emergency recognition and management; f. Patient- and team-centered communication Y-axis: proportion of MS3s or MS4s **indicates statistically significant difference (p<0.05) between MS3 and MS4 students at the mostly entrustable level *indicates statistically significant difference (p<0.05) between MS3 and MS4 students at the fully entrustable level. uta: Unable to assess; re: Pre-entrustable; most: Mostly entrustable; fully: Fully entrustable/Milestone 1; outstanding: Outstanding performance/Milestone 2

Across all six assessment domains, there were some evaluations that specified "unable to assess" (uta) as shown in the bar graphs (Figure 1) and there were only a few students who were classified as pre-entrustable. The majority of students in both classes were assessed as most- or fully-entrustable or outstanding.

In four of the six domains, MS3s were generally judged to be less entrustable than MS4s, as expected (Figure 1). For MS3s, a larger proportion was assessed as being at the lower level of mostly-entrustable compared to fully-entrustable. For the domains of H&P (Figure 1a) and communication (Figure 1f), however, the proportions were similar for both most- and fully-entrustable. The inverse was observed for MS4s. For five of the six domains, a larger proportion of students were judged to be at the higher level of entrustability (fully-entrustable). For the domain "ability to formulate a plan" (Figure 1c) the proportion was similar for both most- and fully-entrustable.

When considering the individual levels of entrusability for each of the six domains, one would expect that a larger proportion of MS3s would be assessed as being at the lower level (most-entrustable) compared to MS4s and that MS4s would dominate at the higher level (fully-entrustable). For all six domains, this is what our analysis revealed. At the lower level of most-entrustable, MS3s had a larger proportion compared to MS4s while at the higher level of fully-entrustable, MS4s dominated. At the level of most-entrustable, the difference between classes was statistically significant for four of the six domains while at the level of fully-entrustable, there was a statistically significant difference for emergency recognition/management (Figure 1e) and for communication (Figure 1f). At the level of outstanding, there was little or no difference between the proportions of each class meeting those criteria. None of the observed differences in this category were statistically significant.

The results of the Hiller comparison demonstrated findings similar to those in their paper [9]. In general, MS4s performed at a slightly higher level than MS3s (67.7% vs. 64.8%, respectively; p < 0.01).

Regression analysis (Table 1) demonstrated a small but statistically significant association between the level of entrustability and months since the start of the third year. The amount of variance explained by months since the start of the third year ranged from 5.5% for the differential diagnosis domain to 1.1% for the emergency recognition and management domain.

**Table 1:** Results of regression analysis comparing the level of entrustability to months since the start of year three H&P: History and physical exam skills; DDx: Differential diagnosis

Assessment domain	Coefficient	Standard error	R^2^	p-value
H&P	0.014	0.003	0.022	<0.01
DDx	0.023	0.003	0.055	<0.01
Formulate plan	0.019	0.003	0.041	<0.01
Observation/monitoring	0.019	0.004	0.022	<0.01
Emergency recognition & management	0.167	0.005	0.011	<0.01
Communication	0.015	0.003	0.024	<0.01

## Discussion

This review of NCAT-EM evaluations of MS3s and MS4s at this institution shows that although there is a general trend towards MS4s outperforming MS3s, this is not statistically significant across all assessment domains. Our conclusion is consistent with that of Hiller et al. [9], who found that MS4s outperformed MS3s when comparing assessments across all categories. When we analyzed our data in a similar manner to Hiller et al. [9], we came up with the same result. Our study further explores this difference by looking at each assessment domain individually, which Hiller et al. [9] did not do, and reveals that two domains - emergency recognition and management and communication - are the best individual metrics that differentiate MS3s and MS4s at our institution. It remains to be seen whether this would hold true in a larger multi-center study.

The timing and circumstances of our study were unique, allowing for control of variables that are often difficult or even impossible to control. With the transition from a required MS4 to a required MS3 clerkship, all students from both classes participated in the clerkship simultaneously. This limits the impact of outside variables produced by time such as strains on the healthcare system, variations in the emergency department setting, and differing evaluators, which all have the potential to impact results.

The process of evaluating medical student performance in an objective way faces many challenges, including grade inflation. This is a known challenge with emergency medicine evaluations, including the standardized letter of evaluation (SLOE) used by EM-bound applicants: marked grade inflation is the norm, limiting the ability of these assessment tools to be discriminating [10,12]. This trend is not limited to emergency medicine but is actually seen across multiple specialties [13]. Beskin et al. demonstrated that this effect is mitigated by more experienced evaluators, as SLOEs written by less experienced writers typically had more average scores of “outstanding” and rated more students as “very competitive” compared to more experienced letter writers [14].

There are three important limitations to this study. First, although our sample size was large at 930, our study might be underpowered to detect the difference in performance between the two classes. Second, we considered that evaluators may not be rating students in a way that truly reflects the performance differences they see on shift due to patterns of evaluation inflation. Faculty and residents receive annual student assessment training under the direction of the Office of Medical Education, which minimizes but does not completely remove inter-rater variance. Rotating students in this institution spend time working alongside both residents and faculty; consequently, 349 out of 931 evaluations were completed by residents whose evaluations may be less discriminating [14].

Another important limitation of this study is the inability to blind evaluators to the level of training of the medical students they work with. The training level is frequently included in the introduction of a medical student to the residents and attendings they work with so that reasonable expectations can be set according to the student’s level of training. MS4s are expected to have more autonomy, allowing them to demonstrate skills, or lack thereof, while expectations for the performance of MS3s are lower. This may result in bias favoring MS4s. These are confounding factors that could warrant further study.

## Conclusions

Our study affirms the findings of the original Hiller et al. study, that the NCAT-EM reliably differentiates between MS4s and MS3s, with MS4s outscoring MS3s globally. Ours is the first study analyzing MS3 versus MS4 performance based on the individual assessment domains within the NCAT-EM. We discovered significant performance differences in two of six domains favoring MS4s over MS3s. This study provides further confirmation for current emergency medicine clerkship directors that the NCAT-EM is a reliable student assessment tool.

## Figures and Tables

**Figure 2:**
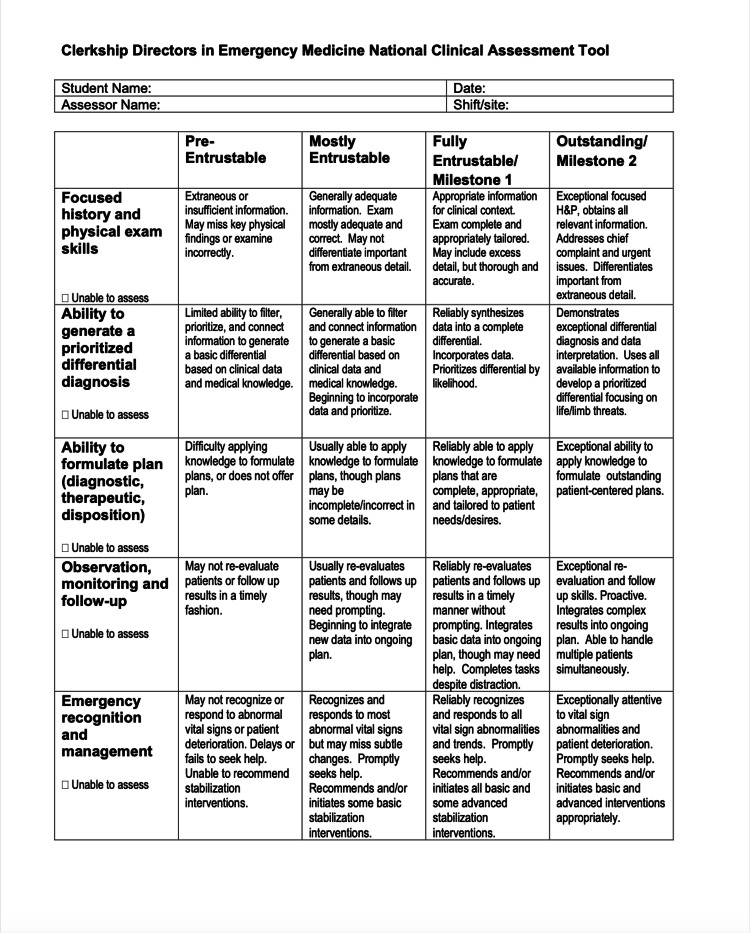
NCAT EM page 1 NCAT-EM: National Clinical Assessment Tool for Medical Students in the Emergency Department

**Figure 3:**
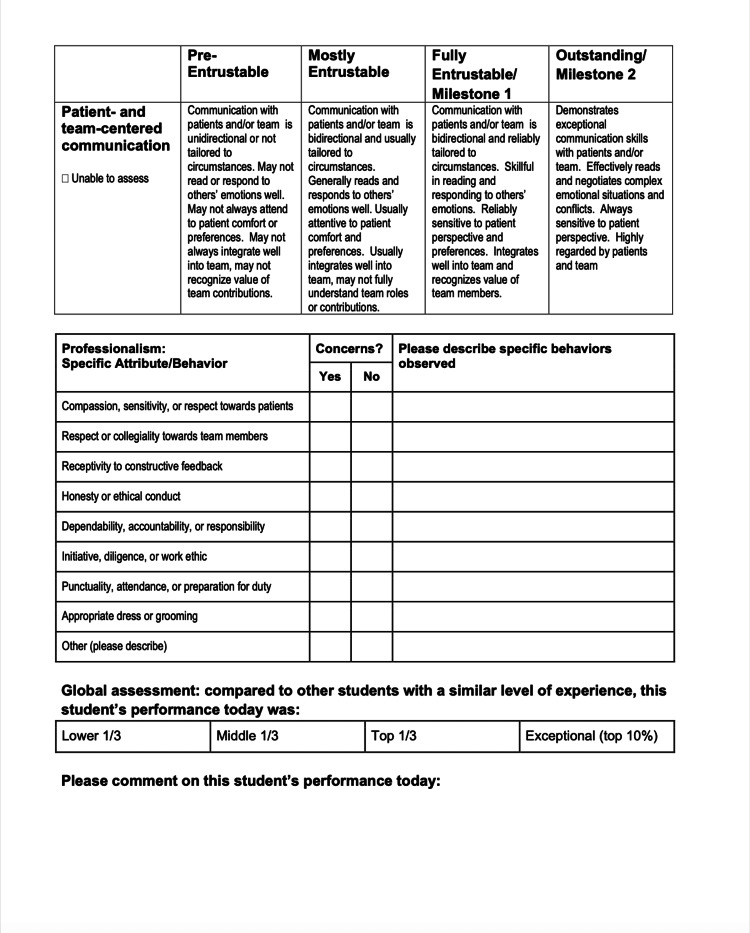
NCAT-EM page 2 NCAT-EM: National Clinical Assessment Tool for Medical Students in the Emergency Department

## Appendices

**Table 2:** Chi-square analysis of MS3 vs. MS4 performance * (p<0.05) MS3: Third-year medical students; MS4: Fourth-year medical students

History and physical			
	MS3	MS4	p-value
Unable to assess	0.009	0.0082	0.99
Pre-entrustable	0.0313	0.0049	0.798
Mostly entrustable	0.3813	0.3071	0.18
Fully entrustable	0.3844	0.4811	0.07
Outstanding	0.1938	0.1987	0.94
Formulating a differential diagnosis			
	MS3	MS4	p-value
Unable to assess	0.0125	0.0066	0.93
Pre-entrustable	0.0531	0.0082	0.66
Mostly entrustable	0.4625	0.3563	0.04
Fully entrustable	0.3469	0.4483	0.07
Outstanding	0.125	0.1806	0.42
Formulating a plan			
	MS3	MS4	p-value
Unable to assess	0.0031	0.0115	qns
Pre-entrustable	0.0375	0.0016	0.85
Mostly entrustable	0.5406	0.4122	0.009*
Fully entrustable	0.3094	0.4187	0.058
Outstanding	0.1094	0.156	0.5
Observation, monitoring, and follow-up			
	MS3	MS4	p-value
Unable to assess	0.075	0.0706	0.95
Pre-entrustable	0.0438	0.0033	0.78
Mostly entrustable	0.3938	0.3202	0.18
Fully entrustable	0.3156	0.4007	0.14
Outstanding	0.1719	0.2053	0.6
Emergency recognition and management			
	MS3	MS4	p-value
Unable to assess	0.2281	0.2151	0.83
Pre-entrustable	0.0094	0	qns
Mostly entrustable	0.3875	0.2594	0.02*
Fully entrustable	0.2375	0.3744	0.03*
Outstanding	0.1375	0.1511	0.83
Communication			
	MS3	MS4	p-value
Unable to assess	0.0034	0.0128	qns
Pre-entrustable	0.0168	0	qns
Mostly entrustable	0.3658	0.2436	0.04*
Fully entrustable	0.3356	0.4615	0.03*
Outstanding	0.2785	0.2821	0.95
